# Supplementary material for: Role and dynamics of vacuolar pH during cell-in-cell mediated death
Source: Cell Death Dis. 2021 Jan 22;12(1):119. doi: 10.1038/s41419-021-03396-2 (PMC7822940; doi:10.1038/s41419-021-03396-2)
Supplement: Supplementary file 1 — Supplementary Figure Legends [file 41419_2021_3396_MOESM1_ESM.docx]

Supplemental Data

Supplemental Figures

**Fig. S1** Keima-based measurement of **the lysosomal pH and cellular pH.** **(A)** Representative images for four keima overexpressed cell lines (550 nm, 440 nm), that were stained with LysoTracker (purple). Scale bar, 20 μm. **(B, C)** The lysosomal pH (B) and cellular pH (C) for four keima overexpressed cell lines. **(D)** The ratio of inner cell fates (live/dead) for four cell lines.

**Fig. S2** **The change of pH value and the entotic death time**. **(A-C)** The change of pH value per hour (A), the cellular pH on death moment (B) and death time (from internalization to cell death) (C) for dead inner cells in MCF7, MCF10A, MDA-MB-231/E and SW480 /E, respectively. **(D-G)** The rate of pH change was negatively correlated with the death time of inner cells in MCF7 (D), MCF10A (E), MDA-MB-231/E (F) and SW480 /E (G), respectively.

**Fig. S3** **The pH dynamics during the CICs-mediated death**. **(A)** The time-lapse images showed keima signal by two channels (550 nm and 440 nm) in one CICs from MCF10A cells. Scale bar, 10 μm. **(B)** The time-lapse images showed keima signal changes in two CICs from MCF7 cells. Blue and white arrows indicate live and dead inner cells respectively. Scale bar, 10 μm. **(C)** Quantification of the cellular pH for non-CIC cells (green) and outer cells of CICs (blue). **(D, F)** Changes in vacuolar pH before and after internalization. **(E, G)** Quantification of the cellular pH for non-CIC cells (dark green), live inner cells (green dots) and dead inner cell (red).

**Fig. S4** **The analysis of vacuolar acidification along with LC3 lipidation**. **(A-C)** Time course-based plotting of vacuolar pH in related to LC3 lipidation during the entosis, including LC3 lipidation before and after entotic death (A), LC3 lipidation after entotic death (B) and LC3 lipidation for live inner cells (C). **(D)** The ratio of LC3 lipidation in three situations of inner cell fate. n (left to right) = 81, 10, 34, respectively. **(E, F)** Time course-based plotting (E) and vacuolar pH (F) in related to LC3 lipidation during the entosis.

**Fig. S5** **The vacuolar pH and cell fates of CICs treated with lysosome inhibitor.** **(A-D)** The distribution of vacuolar pH upon treatments with different concentration of EN6 (A), NH_4_Cl (B), CQ (C) and ConA (D), respectively. The pH value was divided into three level: pH 3-4 (low, red), pH 5-6 (middle, yellow) and pH 7-8 (high, green). **(E-H)** The ratio of inner cell fates (live/dead) for MCF7 cells upon treatments with different concentration of EN6 (E), NH_4_Cl (F), CQ (G) and ConA (H) n (left to right) = 16, 25, 25, 23 for (A, E); 16, 30, 21, 26, 26 for (B, F); 16, 10, 20, 20 for (C, G); 16, 22, 23, 23, 18 for (D, H).

**Fig. S6** **ATG5/7 and V-ATPase are required for vacuolar acidification and entotic cell death.**  **(A, B)** The relative mRNA level of ATG5 (A) and ATG7 (B) for MCF10A cells transfected with the corresponding siRNAs. **(C-F)** The percentage of LC3 recruitment onto vacuole (C), the pH of vacuoles containing live or dead inner cells (D), inner cell fates (E), and the ways inner cells died (entotic or apoptotic) (F) for MCF10A cells transfected with the corresponding siRNAs indicated. n (left to right) = 72, 77, 69, 69, 69 for (C); 21, 16, 50, 18, 46, 23, 34, 14, 23, 21 for (D); 37, 22, 22, 19, 22 for (E); 37, 22, 22, 19, 22 for (F). ** *P*<0.01; ****P*<0.001. **(G)** The relative mRNA level of ATP6V0A2 and ATP6V1A for MCF10A cells transfected with the corresponding siRNAs. **(H-J)** The pH of vacuoles containing live or dead inner cells (H), inner cell fates (I), and the ways inner cells died (entotic or apoptotic) (J) for MCF10A cells transfected with siRNAs of ATP6V0A2 and ATP6V1A. n (left to right) = 20, 16, 30, 12, 28, 23 for (H); 72, 68, 69 for (I); 37, 23, 22 for (J). * *P*<0.05; ** *P*<0.01; ****P*<0.001.

Supplemental Movies

**Movie. S1** The time-lapse microscopy movies showed keima signal changes in one CICs from MCF10A cells.

**Movie. S2** The time-lapse microscopy movies showed keima signal changes in two CICs from MCF7 cells.

**Movie. S3** Time-lapse microscopy movies of transient recruitment of GFP-LC3 (purple) to the entotic vacuole prior to inner cell death.

**Movie. S4** Time-lapse microscopy movies of vacuolar GFP-LC3 (purple) recruitment following inner cell apoptosis.

**Movie. S5** Time-lapse microscopy movies of vacuolar GFP-LC3 (purple) recruitment following inner cell entosis.

**Movie. S6** Time-lapse microscopy movies of vacuolar GFP-LC3 (purple) recruitment for alive inner cells.
