# Supplementary figures and images for: Role and dynamics of vacuolar pH during cell-in-cell mediated death
Source: Cell Death Dis. 2021 Jan 22;12(1):119. doi: 10.1038/s41419-021-03396-2 (PMC7822940; doi:10.1038/s41419-021-03396-2)

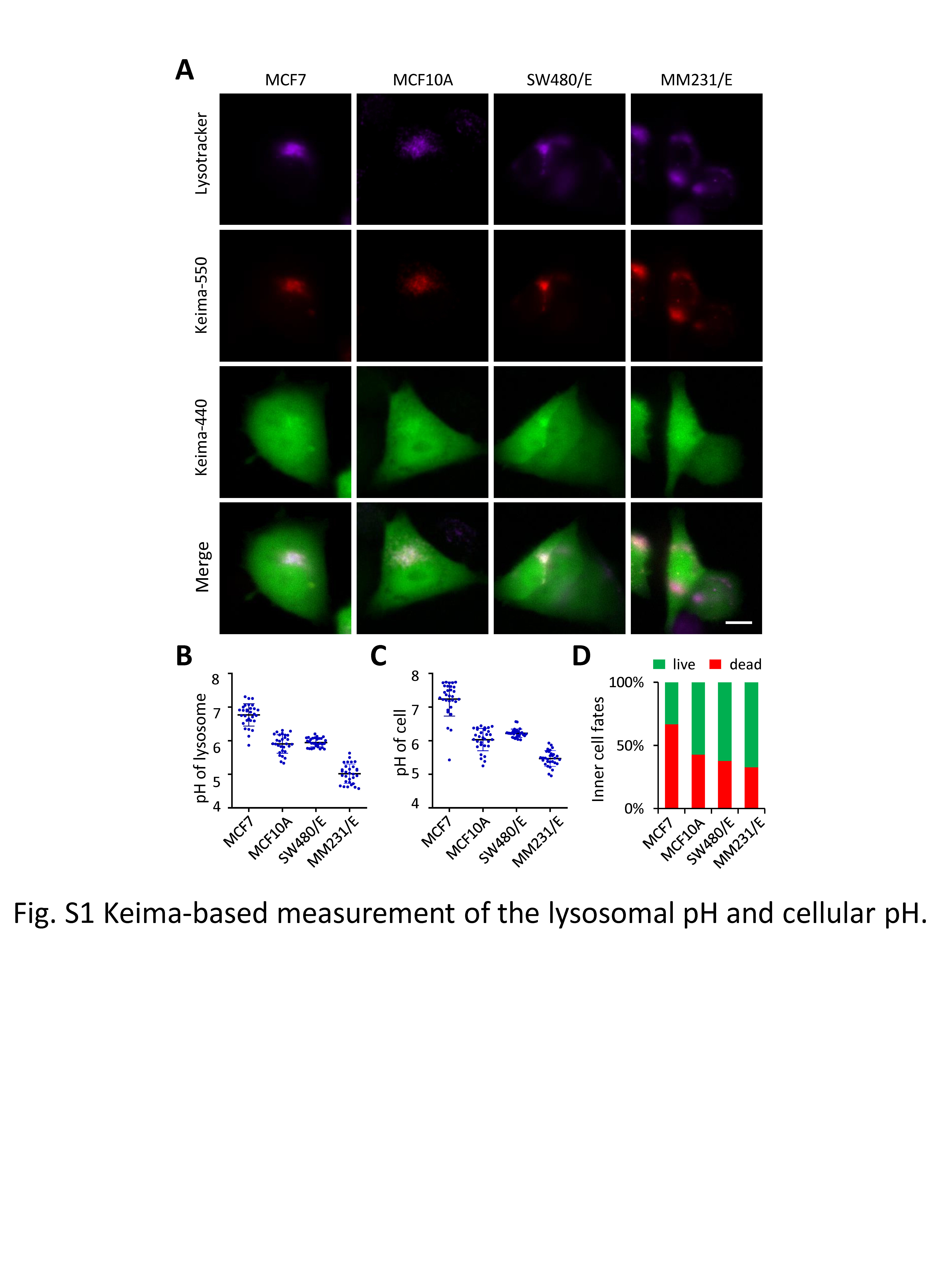

Supplement: Supplementary file 2 — Figure S1 [file 41419_2021_3396_MOESM2_ESM.tif]

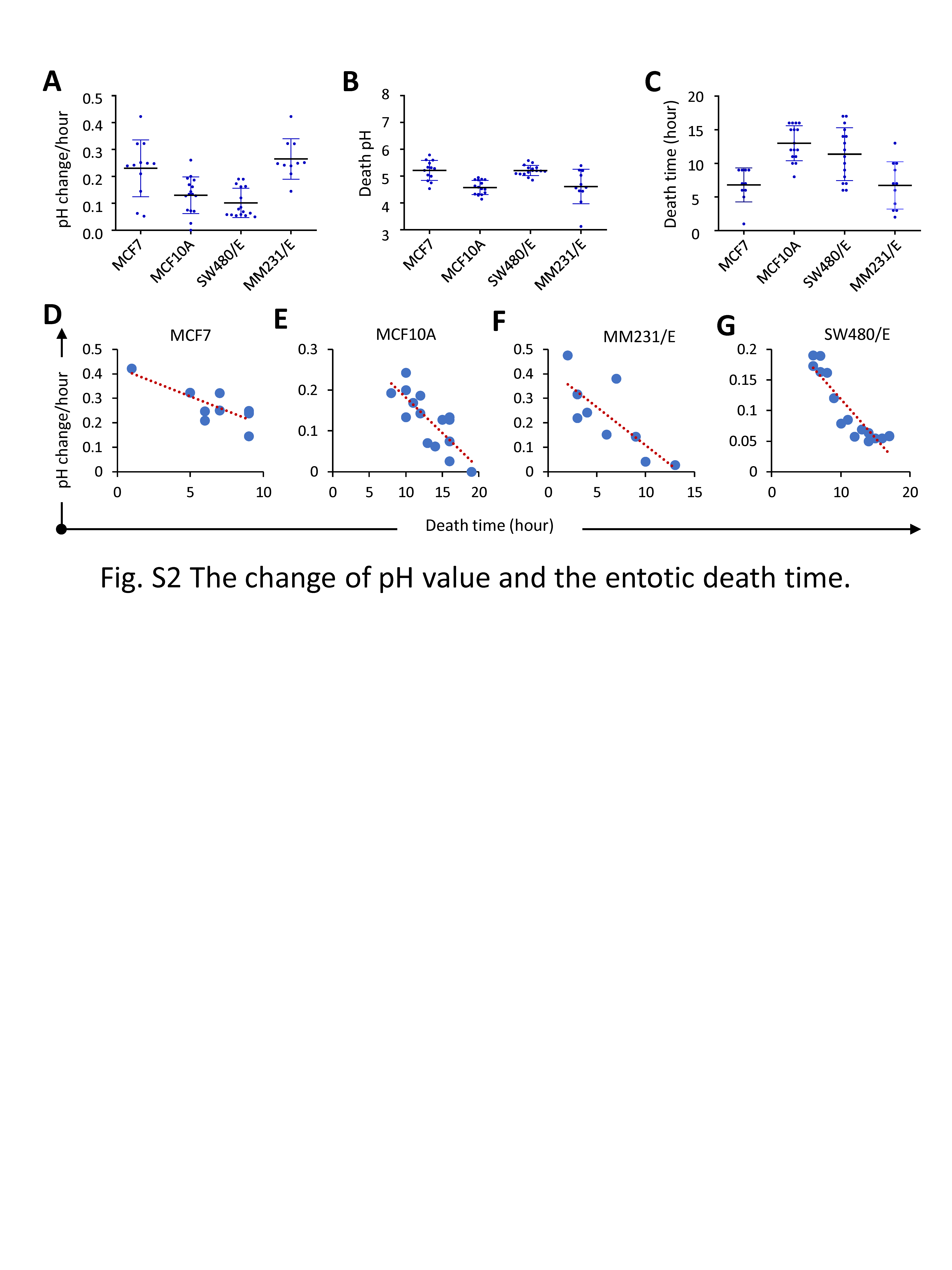

Supplement: Supplementary file 3 — Figure S2 [file 41419_2021_3396_MOESM3_ESM.tif]

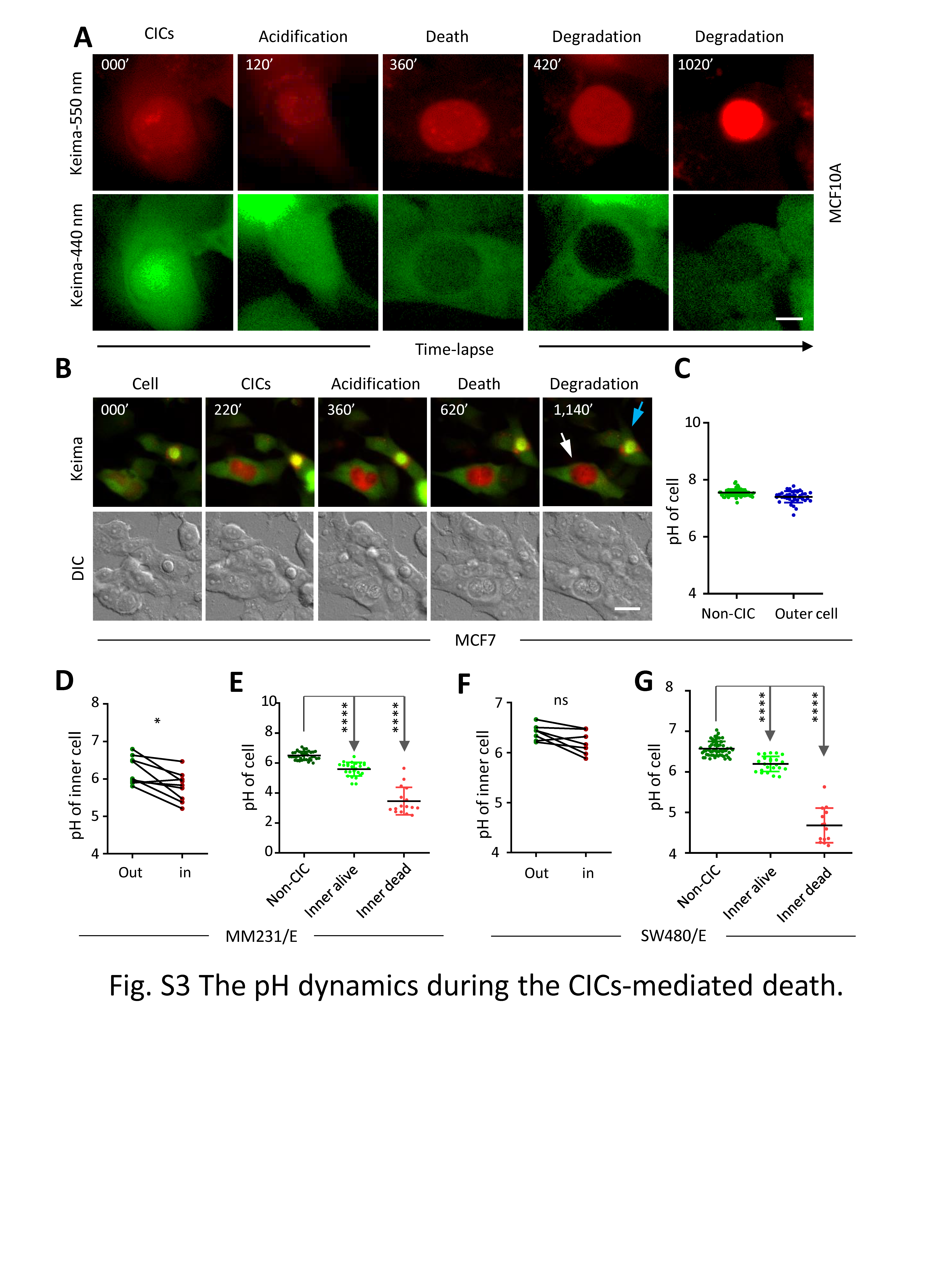

Supplement: Supplementary file 4 — Figure S3 [file 41419_2021_3396_MOESM4_ESM.tif]

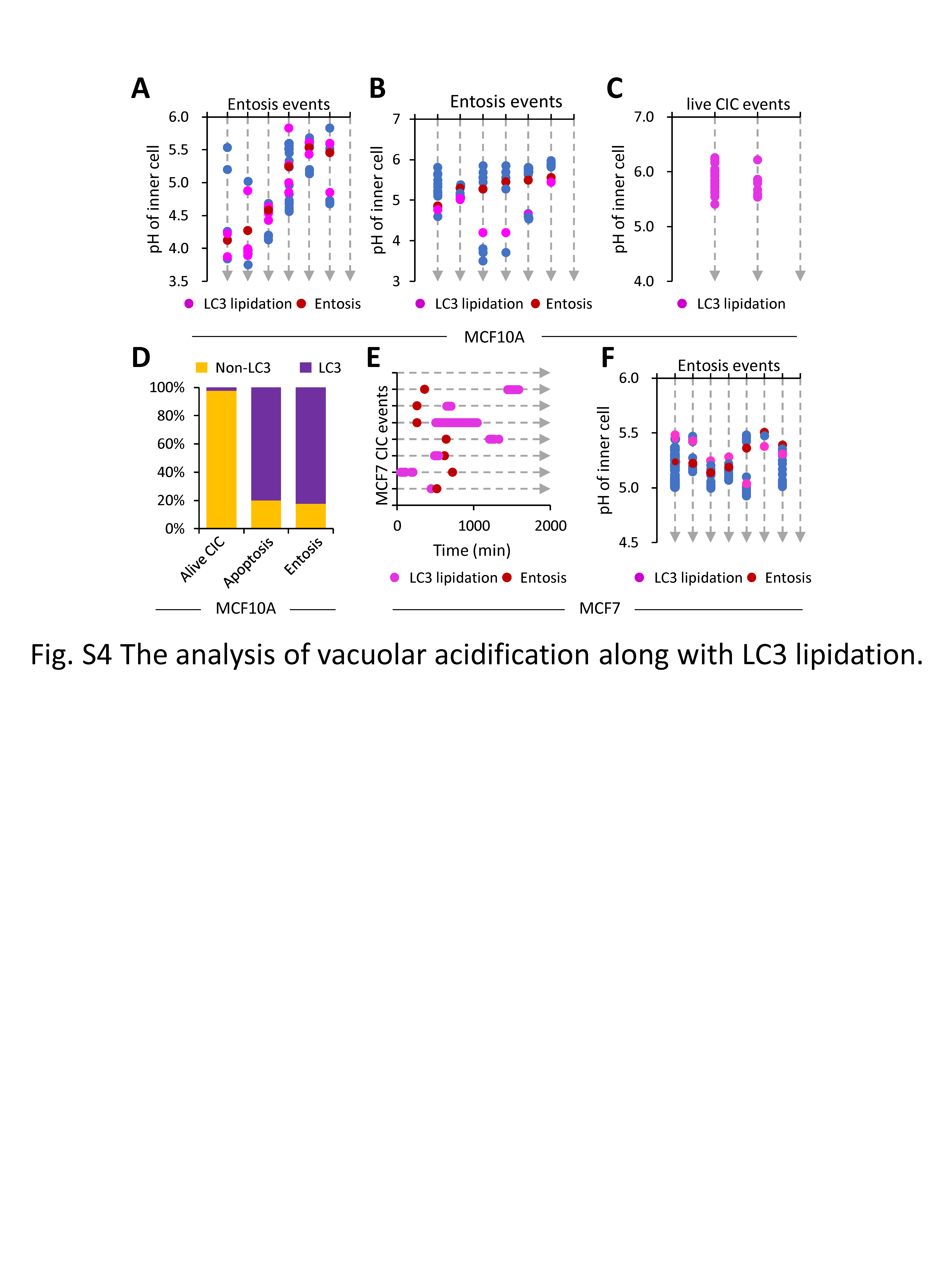

Supplement: Supplementary file 5 — Figure S4 [file 41419_2021_3396_MOESM5_ESM.tif]

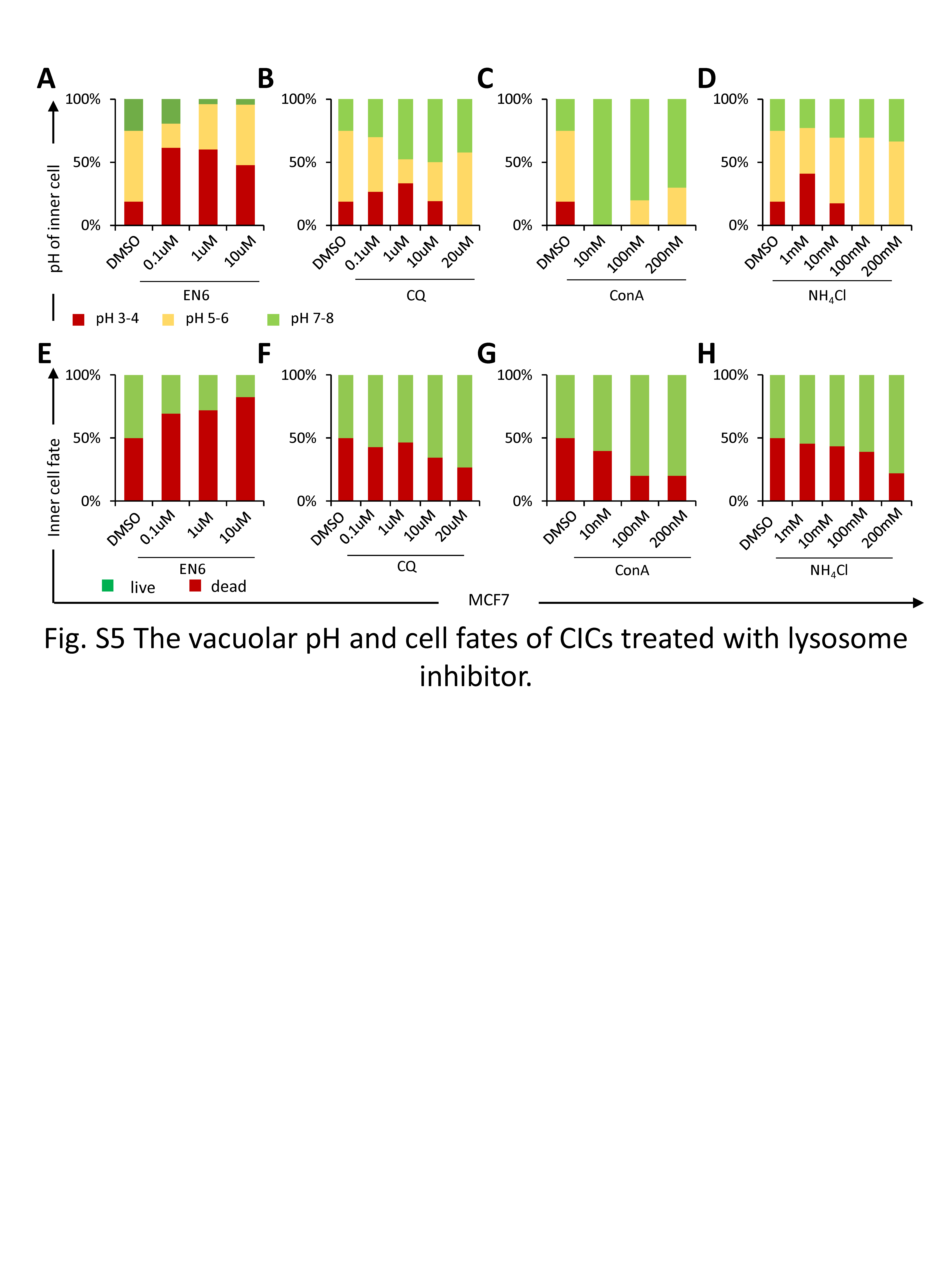

Supplement: Supplementary file 6 — Figure S5 [file 41419_2021_3396_MOESM6_ESM.tif]

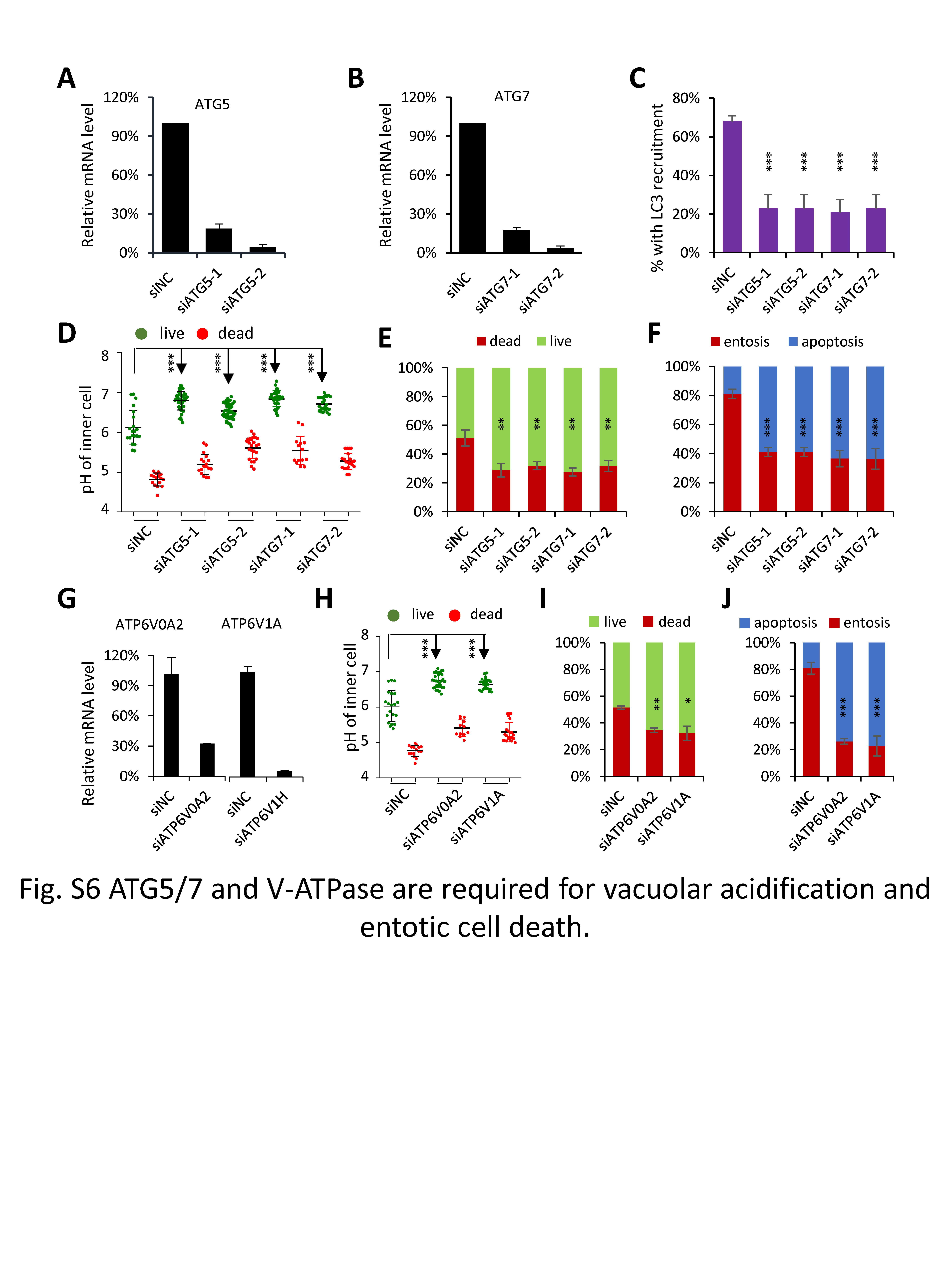

Supplement: Supplementary file 7 — Figure S6 [file 41419_2021_3396_MOESM7_ESM.tif]
